# A remarkable permeability enhancement of Ni_1−x_Zn_x_Fe_2_O_4_ (x = 0.65 and 0.70), using a multi-compound calcined additive

**DOI:** 10.1038/s41598-024-64016-5

**Published:** 2024-06-06

**Authors:** S. Shatooti, M. Mozaffari

**Affiliations:** Department of Physics, Faculty of Physics, University of Isfahan, Isfahan, 81746-73441 Iran

**Keywords:** Ni–Zn ferrite, Solid-state reaction, Sintering process, Multi compound calcined additive (MCCA), Initial permeability, Engineering, Materials science

## Abstract

In this study, entanglement of composition, additive and/or sintering conditions and their effects on magnetic properties of soft ferrites, nickel zinc spinel ferrites (Ni_1−x_Zn_x_Fe_2_O_4_, x = 0.65 and 0.70) which were prepared via conventional solid-state reaction method investigated. Also an equiponderant calcined mixture of Bi_2_O_3_, CaO, CeO_2_, SiO_2_, Al_2_O_3_, Y_2_O_3_ and nanotitania was mixed thoroughly and used as a multi-compound calcined additive (MCCA). Calcined ferrite powders were crushed, dry and wet milled, dried, mixed with different amounts of MCCA (0.0, 0.5, 1.0, 1.5 and 2.0 wt%), formed in toroidal shapes and finally sintered at different temperatures, from 1150 up to 1360 °C for 3 h. X-ray diffraction assessment confirmed formation of the single phase cubic spinel structures. Initial permeability and Q-factor spectra of the toroids were obtained from 0.1 to 1000 kHz, using an LCR meter. The results show that initial permeability of each sample has a maximum and addition of MCCA to the ferrites leads to a marvelous increase in permeabilities. Additionally, MCCA decreases the optimum sintering temperature too. The optimum amounts of additive were 1.0 and 0.5 wt% for the x = 0.65 (μ′ = 492, T_s_ = 1280 °C) and x = 0.70 (μ′ = 478, T_s_ = 1320 °C), respectively. Permeability spectra illustrate that utility zone of the Ni_0.35_Zn_0.65_Fe_2_O_4_ and Ni_0.3_Zn_0.7_Fe_2_O_4_ are both less than 100 and 10 kHz, respectively. The results represent that there is a strong entanglement between composition, additive and/or sintering conditions. It can be concluded the MCCA added Ni_0.35_Zn_0.65_Fe_2_O_4_, is suitable for application in the switching power supplies.

## Introduction

Nowadays, ceramics due to their stronger ionic or covalent bonding than metals have attracted a great deal of attentions and applications in various fields such as; electronic industry, high temperature superconductors, automotive engines, and medical implants^1,2^. Ferrites as non-conductive ceramics have been investigated and hired in electronic and electric devices due to high electrical resistivity, high chemical stability, and their applicable frequency-dependent permittivity and permeability in the radio frequency (RF) region^3,4^. Spinel ferrites with cubic crystal structure, with the fd$\overline{3 }$m space group, are expressed by the general chemical formula MFe_2_O_4_, where M is a divalent cation such as Fe^2+^, Mn^2+^, Ni^2+^, Zn^2+^, Co^2+^, … or combinations of the different cations with a resultant valence of two. They have been extensively utilized in different field of technology such as electronic and communicating devices, catalyst, drug delivery, gas sensors, magnetic hyperthermia and etc.^5–8^.

At low frequencies (< 100 kHz), ferrites are used as core of inductors in low pass LC filters. Due to their high permeability and high resistivity, which in turns result in low eddy current loss at high frequencies (> 100 kHz), ferrites have been extensively used in electromagnetic devices e.g., antenna rods, cores in chock coils, inductors and transformers^9–11^. Thereupon, Ni–Zn ferrites as mixed spinel soft-magnets are appropriate candidates for applications from MHz to GHz frequencies, due to their high resistivity^12,13^. In addition, it has capability to absorb electromagnetic radiation at high frequencies, which is representing it as an appropriate material for magnetic absorbers in electronic devices^14^.

Ni–Zn ferrites with various amounts of zinc content and different dopants, prepared via different synthesis routes such as; sol–gel, co-precipitation, hydrothermal and conventional ceramic technique in diversity of conditions have been perused and reported^15–19^. The magnetic and electrical properties of Ni–Zn ferrite like to other ferrites is considerably dependent on cation distribution and microstructure, which in turn depend on preparation path and starting materials^20,21^.

In the ceramic industry, the conventional ceramic technique is a common synthesis route, because of availability and low cost staring reagents. In this route, proper stoichiometric amounts of oxides or carbonates of metals, as raw materials, are thoroughly wet and/or dry mixed and calcined to get desired phase. The calcined powders are crushed and milled to get submicron particles and formed into desired shapes. After that the shaped sample is sintered at relatively high temperatures to get final density and microstructure^22^. Solid-state sintering occurs when the compacted powders (green body) are heated at temperatures below the melting points of each component. Sintering is a key step in the solid-state reaction in the preparation and production of ferrites, which it in turns affect the microstructure, electric and magnetic properties of the final products^23^.

Additionally, in the so-called additive sintering a small amount of insoluble substance or a combination of substances are used, which influence magnetic and electrical properties and/or microstructure of ferrites by different mechanisms^24–26^. In high frequency ferrites’ preparation, it is required to use at least one additive. There are three categories of additives as follow: first group comprises additives which segregate on the grain boundaries and affect the grain-boundary resistivity, e.g. SiO_2_ and CaO. The second ones include additives that improve microstructure during sintering via liquid phase formation, such as Bi_2_O_3_. The third group encompasses additives which are solved into the grains and modify the magnetic properties, e.g. TiO_2_^27^.

In this work, the effects of a multi-compound calcined additive (MCCA), including equiponderant CaO, CeO_2_, SiO_2_, Al_2_O_3_, Y_2_O_3_, nanotitania and Bi_2_O_3_, in which the last one is doubled in weight, on the sintering temperatures and permeabilities of Ni_1−x_Zn_x_Fe_2_O_4_ (x = 0.65 and 0.70) were investigated and compared with those of the additive-free samples.

## Experimental

### Synthesis procedure

Fe_2_O_3_ (from a domestic source with minimum purity of 99.5%), NiO (with minimum 75% Ni content, Merck Co., Germany), ZnO (99.5%, Merck Co., Germany), Bi_2_O_3_ (99%, Merck Co., Germany), CaO (99.9%, Sigma Aldrich), CeO_2_ (99% from Scharlau), SiO_2_ (from a domestic source with minimum purity of 99%), nanotitania (99%, Teconan), Y_2_O_3_ (99%, Riedel-Deltaen) and Al_2_O_3_ (99.99%, Merck Co., Germany) were used as starting materials.

To investigate the effect of a multi compound additive on the permeability and sintering temperature of the Ni_1−x_Zn_x_Fe_2_O_4_ spinel ferrites, a mixture of the same weight of CaO, CeO_2_, SiO_2_, nanotitania, Y_2_O_3_, Al_2_O_3_ and Bi_2_O_3_, in which the last one was doubled in weight, was ball milled in an agate vial and balls for an hour and then calcined at 1100 °C for 3 h, which is named MCCA.

Ni_1−x_Zn_x_Fe_2_O_4_ (x = 0.65 and 0.70) powders were prepared via conventional ceramic technique. The appropriate stoichiometric amounts of the raw reagents were weighed according to the following chemical reaction:

1 $$\left( {{1} - {\text{x}}} \right){\text{ NiO}} + {\text{x ZnO}} + {\text{Fe}}_{{2}} {\text{O}}_{{3}} \to {\text{Ni}}_{{{1} - {\text{x}}}} {\text{Zn}}_{{\text{x}}} {\text{Fe}}_{{2}} {\text{O}}_{{4}} ,$$

where x = 0.65 and 0.70. The reagents were wet mixed in ethyl alcohol, using an agate vial and balls for 10 h in a planetary ball mill (FRITSCH P6) to get homogeneous mixtures. The mixed powders were dried at room temperature to evaporate the alcohol content. The dried powders were calcined at 1050 °C for 3 h in the air free cooling to room-temperature. The calcined powders were crushed and wet milled for 10 h, in the same conditions as mixing stage to get fine powders. After air drying, different amounts of additive (0.0, 0.5, 1.0, 1.5 and 2.0 wt%) were added to the crushed calcined powders and mixed in an agate vial for an hour using a vibrating ball mill (RETSCH, MM2 mixer mill) to obtain homogeneous mixture. Then, mixed powders were blended with 10 wt% polyvinyl alcohol (5% PVA solution) as a binder, and were formed into toroids (D_out_ = 32 mm, D_in_ = 24 mm and about 5 mm in height) using a hydraulic pressing machine under 14 ton/cm^2^ pressure. The toroids were sintered at various temperatures (T_s_), from 1150 to 1360 °C for 3 h. Heat treatment flowchart sintering is shown below:


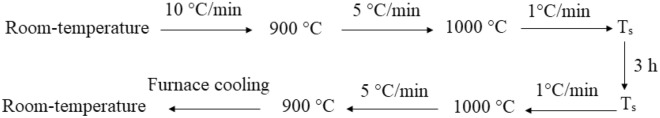


### Characterization

Phase identification of the samples was carried out with an x-ray diffractometer (BRUKER, D8 ADVANCE model) equipped with Cu tube (Cu-Kα radiation, λ = 1.5406 Å) at a scanning rate of 0.04°/s between 20 and 80 (2θ) degrees. X’pert High Score Plus software was used to characterized XRD patterns. Mean crystallite sizes of the powders and their strains of the crystallites were obtained by Williamson-Hall method^28^:

2 $$\upbeta \cos\uptheta = \frac{{0.9\uplambda }}{{\text{D}}} + 2\upepsilon \sin\uptheta ,$$

where λ is x-ray wavelength, θ is the Bragg angle, β is the full width at half-maximum (FWHM) that is ascribed to broadening of the crystallite size and strains, D is the mean crystallite size and ε is the strain. To obtain D and ε, a line was fitted in the $\beta \cos \theta$ with respect to $\sin \theta$ and based on the Williamson-Hall formula, y-intercept is $\frac{{0.9\uplambda }}{{\text{D}}}$ and its slope equals to 2ε.

As the crystal structure of the samples is cubic, their lattice constants were calculated by the following relation^29^:

3 $${\text{d}} = \frac{{{\text{a}}^{{2}} }}{{{\text{(h}}^{{2}} {\text{ + k}}^{{2}} {\text{ + l}}^{{2}} {)}^{{1/2}} }},$$

where $a$ is the lattice constant and d is interplanar spacing of the plane with (hkl) Miller indices.

X-ray density of the samples ρ_x-ray_, were calculated using the following formula^30^:

4 $$\uprho _{{{\text{x}} - {\text{ray}}}} = \frac{{{\text{8M}}}}{{{\text{N}}_{{\text{A}}} }}{\text{a}}^{{3}} ,$$

where M is the formula unit weight, $a$ is the lattice constant and N_A_ is the Avogadro's number.

The toroidal shape samples were wrapped by 30 turns (N) enameled copper wire and their R_s_ and L_s_ parameters were measured, using an LCR meter (Fluke, PM6303) in the frequency range of 0.1 to 1000 kHz at low magnetic flux densities. The real part of permeability (μ′) and Q-factor were calculated by the following relations: $\upmu ^{\prime } = \frac{{{\text{L}}{_{\text{s}} {{\text{L}}_{{\text{m}}} }} }}{{{\upmu }{_{0} {{\text{N}}^{2}}} {\text{A}}_{{\text{e}}} }}$ and ${\text{Q}} = \frac{{{\omega L}_{{\text{s}}} }}{{{\text{R}}_{{\text{s}}} }}$, respectively, where ω = 2πf, L_s_ is the series self-inductance, L_m_ and A_e_ are related to the toroids geometry by ${\text{L}}_{{\text{m}}} = \frac{{\uppi \times ({\text{D}}_{{{\text{out}}}} + {\text{D}}_{{\text{in }}} )}}{2}$ and ${\text{A}}_{{\text{e}}} = \frac{{\left( {{\text{D}}_{{{\text{out}}}} - {\text{ D}}_{{\text{in }}} } \right) \times {\text{h}}}}{2}$, D_out_, D_in_ and h are outer diameter, inner diameter and height of the toroids, respectively.

## Result and discussion

### Structural analysis

**Figure 1:**
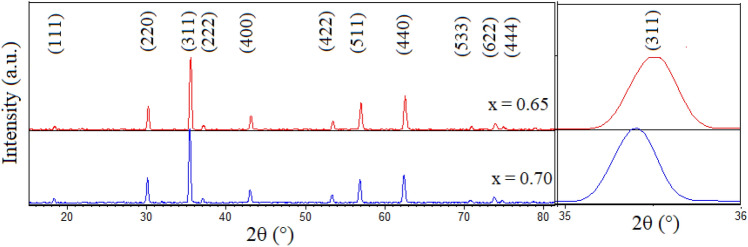
Left: XRD patterns of the Ni_1−x_Zn_x_Fe_2_O_4_ (x = 0.65 and 0.70) powders, calcined at 1050 °C. Right: Expanded (311) diffraction peak.

X-ray diffraction patterns of both calcined powders (x = 0.65 and 0.70) are shown in Fig. 1(left). As can be seen, all detectable peaks are well matched with the standard spinel’s peaks, which are labeled by PDF card No. 08-0234. This illustrates that single phase spinel crystal structures are mainly formed in both powders. Also a comparison between two patterns shows that all diffraction peaks related to the x = 0.70 sample are shifted to lower diffraction angles with respect to those of the x = 0.65 one, which is represented in Fig. 1(Right) clearly. This lead to an increase in lattice parameter based on Vegard′s law^31^, which is due to substitution of larger Zn^2+^ (R_Zn_^2+^: 0.082 nm) ion for the smaller Ni^2+^ (R_Ni_^2+^: 0.072 nm) one in A sites^32^. Calculated lattice parameters, mean crystallite sizes, x-ray densities, lattice strains of the calcined powders and bulk densities of the sintered samples at 1250 °C are tabulated in Table 1.

**Table 1:** Lattice parameters (a), mean crystallite sizes (D), strains (ε), X-ray (ρ_x-ray_) and bulk (ρ_b_) densities.

Sample	a ± 0.001 (Å)	D ± 1 (nm)	ε	ρ_x-ray_ (g/cm^3^)	ρ_b_ (g/cm^3^) for Ts = 1250 °C
Ni_0.35_Zn_0.65_Fe_2_O_4_	8.393	26	0.006	5.365	4.76
Ni_0.3_Zn_0.7_Fe_2_O_4_	8.412	29	0.002	5.337	5.03

### Permeability and Q-factor spectra

**Figure 2:**
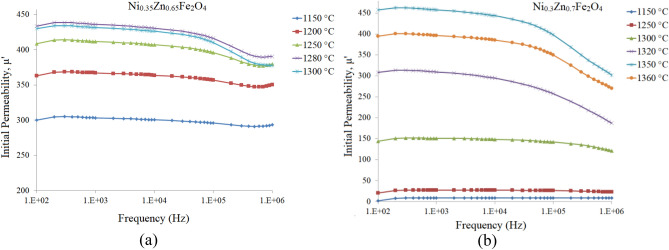
Real part of the initial permeability spectra for additive free (**a**) Ni_0.35_Zn_0.65_Fe_2_O_4_ and (**b**) Ni_0.3_Zn_0.7_Fe_2_O_4_ samples at different sintering temperatures.

Real part of the initial permeability spectra related to the additive free Ni_0.35_Zn_0.65_Fe_2_O_4_ samples (sintered at 1150, 1200, 1250, 1280 and 1300 °C) and that for Ni_0.3_Zn_0.7_Fe_2_O_4_ samples (sintered at 1150, 1250, 1300, 1320, 1350 and 1360 °C) are presented in Fig. 2a and b, respectively.

The permeability of polycrystalline ferrites can be originated from two different magnetizing mechanisms; domain wall motion and spin rotation. The permeability follows:

5 $$\upmu = {1} +\upchi _{{{\text{dw}}}} +\upchi _{{{\text{spin}}}} ,$$

where χ_dw_ and χ_spin_ are magnetic susceptibilities of the domain wall motion and spin rotation, respectively^33^. On the other hand:

6 $$\upchi _{{{\text{dw}}}} = \frac{{3\uppi {\text{DM}}_{{{\text{s}} }}^{2} }}{4\gamma },\quad\upchi _{{{\text{spin}}}} = \frac{{2\uppi {\text{M}}_{{{\text{s}} }}^{2} }}{{\text{K}}}$$

where M_s_ is saturation magnetization, D is mean grain size, γ is domain wall energy and K is effective magnetic anisotropy constant. As domain wall motion is restricted by the grain boundaries, grains growth leads to lesser grain boundaries and then a predominant enhancement in permeability occurs^34–37^.

Although variation of the permeability with respect to frequency for the x = 0.65 sample (Fig. 2a) is negligible at lower sintering temperatures, but at higher ones and higher frequencies (> 100 kHz) decreases slightly. It is due to increase in Eddy current loss which is more important at higher frequencies. This restricts the applicability frequency range of the ferrites^38,39^.

Figure 2b shows permeabilities of the Ni_0.3_Zn_0.7_Fe_2_O_4_ samples, sintered up to 1300 °C are relatively constant, but as can be seen the permeabilities slightly decrease, as sintering temperature increases, at frequencies greater than 100 kHz. On the other words, as sintering temperature increases utility zone decreases, which is due to grain growth^40^.

**Figure 3:**
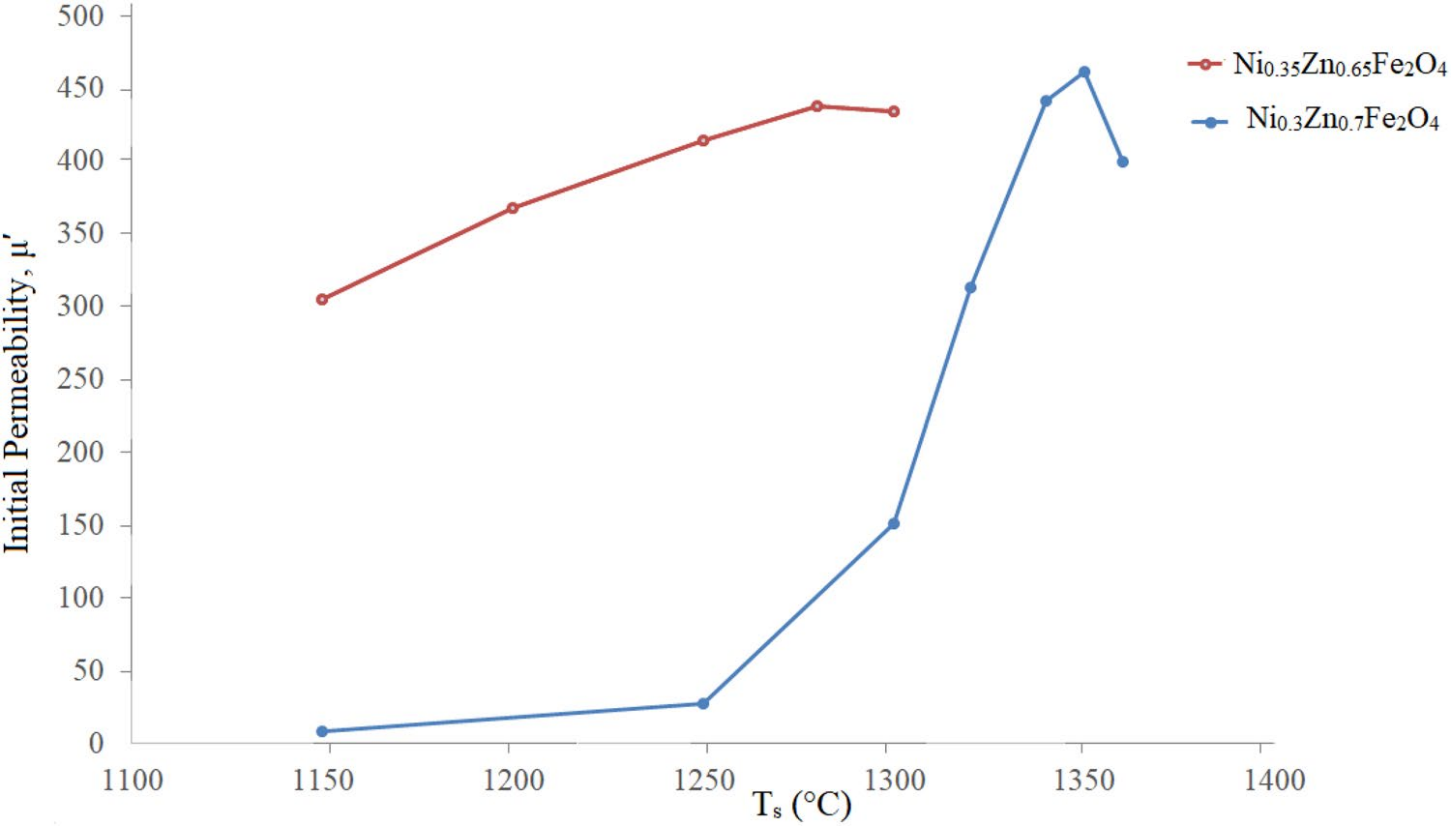
Variations of the real part of initial permeability (@ 1 kHz) of the additive free samples versus T_s_.

To determine the effect of the sintering temperature on real part of permeability, their variations with respect to T_s_ were plotted in Fig. 3 at 1 kHz, which are representing values in the utility zone. As can be seen both series have maxima, which are 438 for the x = 0.65 (sintered at 1280 °C) and 462 for x = 0.70 (sintered at 1350 °C). The increase in permeability by increasing the sintering temperature is due to grain growth and in turn densification. The grain growth causes to decrease pores, which are domain walls' motion inhibitor. Thereby the pinning wall becomes fewer and walls move easier, which leads to higher permeability. Additionally, by increasing sintering temperature, residual stresses reduce, which in turn result in magnetic anisotropy reduction. Therefore domain wall movement facilitates and the initial permeability increases^41^.

As mentioned above, there is an optimum sintering temperature for each series at which the permeability is maximum. This behavior is depending on sample densification in the course of sintering. As sintering temperature increases, some Fe^3+^ ions reduce to Fe^2+^ ones and causes to make oxygen vacancies to keep charge neutrality. As Fe^2+^ ions diffuse faster than Fe^3+^ ones, thereupon grain growth turns up, which results in an increase in densification with larger grains^42^. Above optimum sintering temperature the permeability decreases which can be related to the increase in the pores within the grains in spite of increasing grain size.

**Figure 4:**
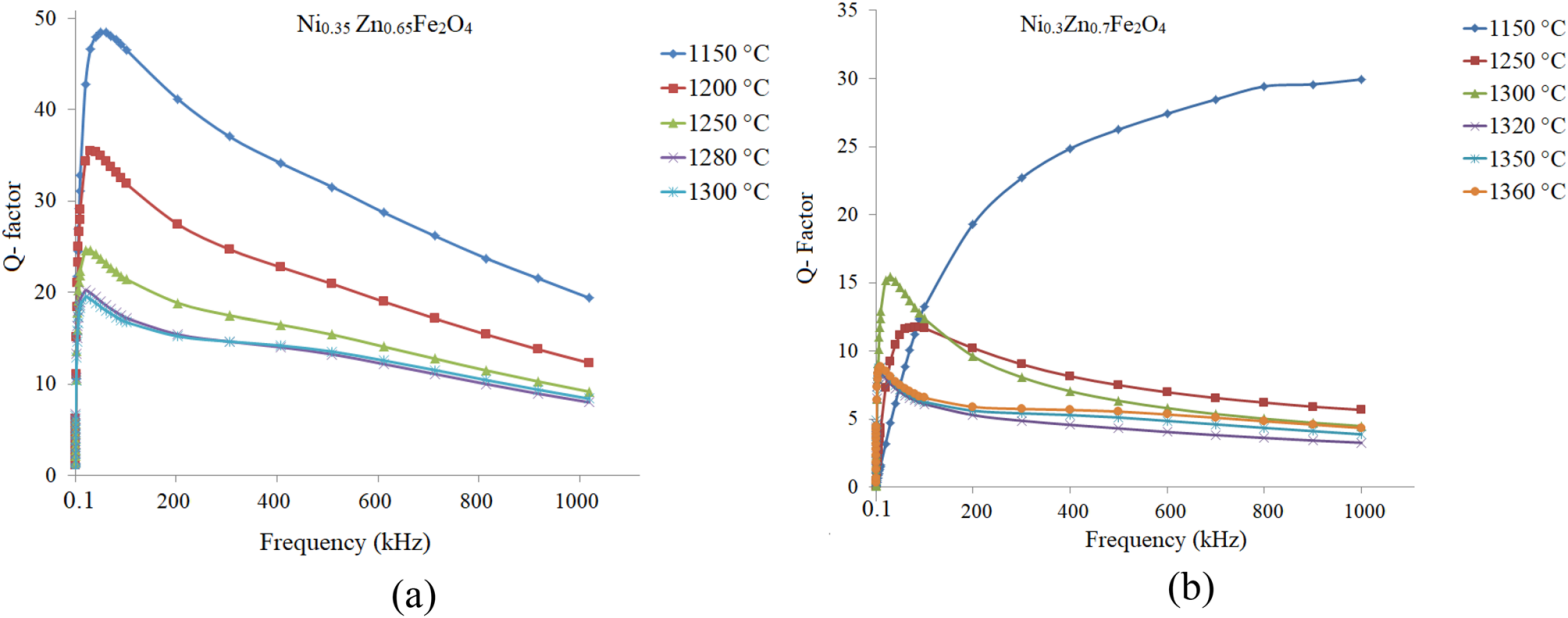
Q-factor spectra of the additive free samples, sintered at different temperatures: (**a**) Ni_0.35_Zn_0.65_Fe_2_O_4_, and (**b**) Ni_0.3_Zn_0.7_Fe_2_O_4_.

Figure 4a and b show quality factors (Q-factors) spectra of the additive free sintered samples at various sintering temperature. As can be seen Q-factor of all samples raised rather rapidly with frequency showing a sharp peak and then fell, except that sintered at 1150 °C (x = 0.70). Highest value of Q-factor for both series achieved at T_s_ = 1150 °C. The highest Q-factor of the samples at this sintering temperature may be ascribed to smaller grain size and lesser defects inside the grains than the samples sintered at higher temperatures.

### The effect of additive on permeability, Q- factor and sintering temperature

**Figure 5:**
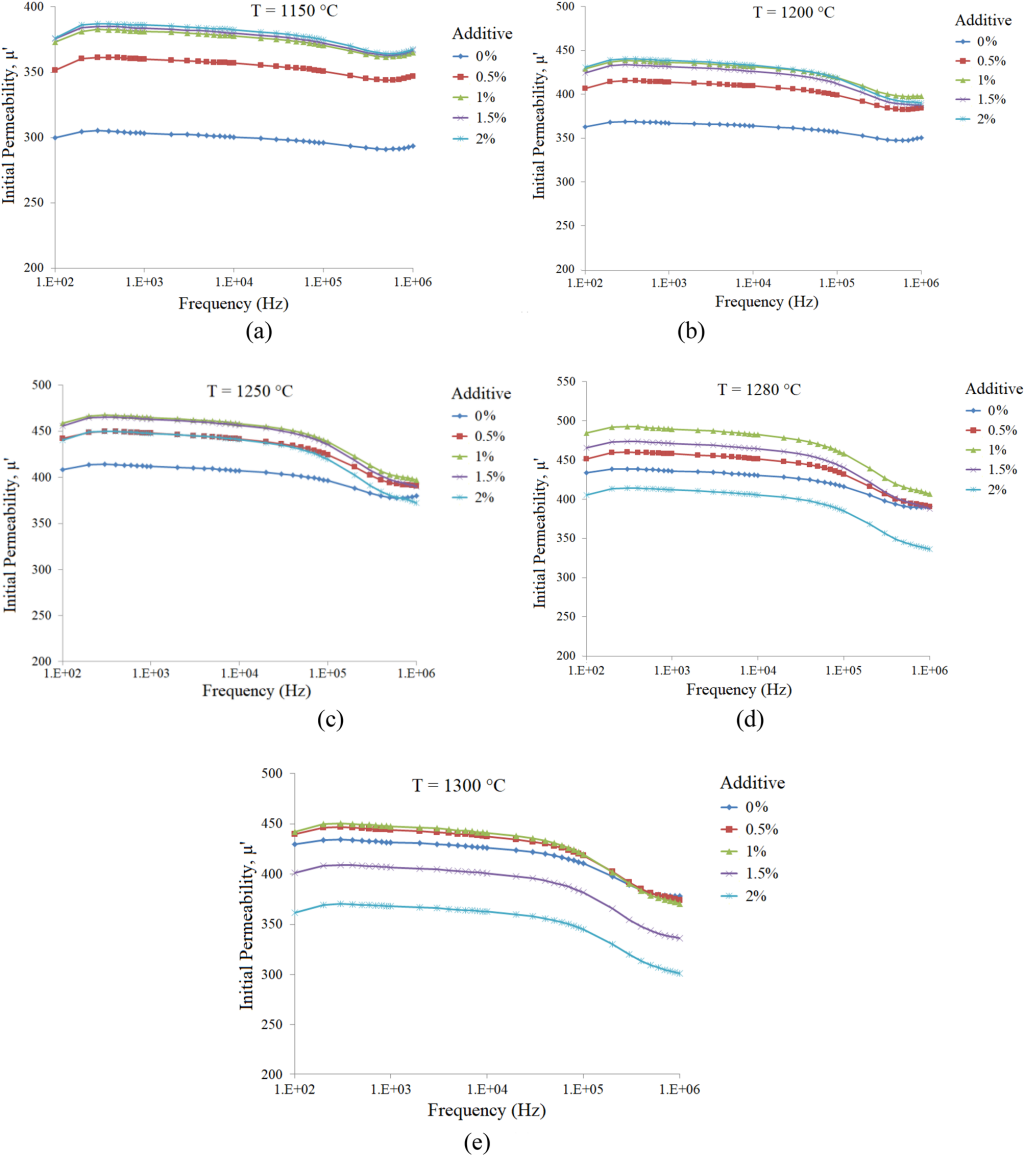
Initial permeability spectra of the Ni_0.35_Zn_0.65_Fe_2_O_4_ samples with additive (0.0–2.0 wt%) and sintered at various temperatures: (**a**) 1150, (**b**) 1200, (**c**) 1250, (**d**) 1280 and (**e**) 1300 °C.

**Figure 6:**
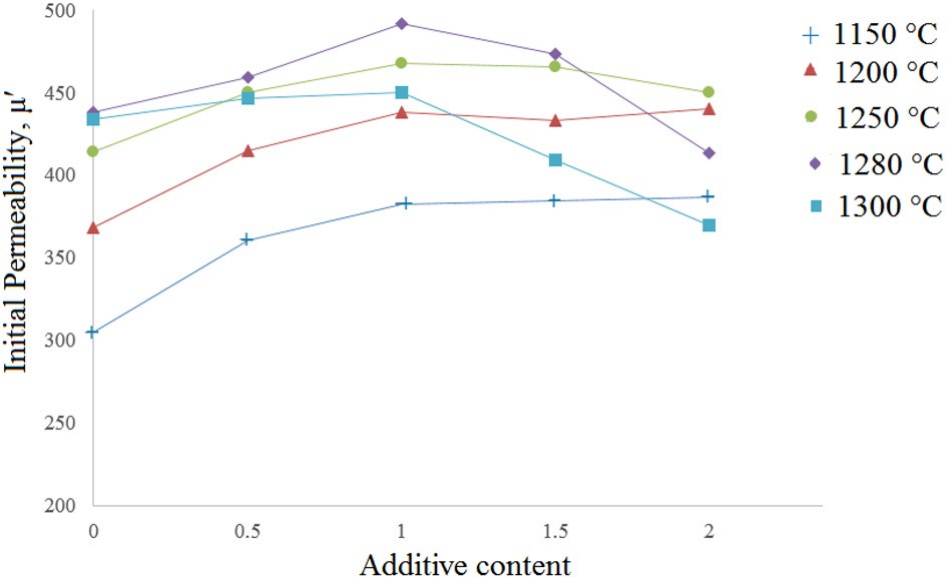
The variation of initial permeability (@ 1 kHz) of the Ni_0.35_Zn_0.65_Fe_2_O_4_ samples with respect to additive contents, sintered at various temperatures as labeled on each curve (The lines were added to guide eye).

Figure 5a–e illustrate the initial permeability spectra of the x = 0.65 samples with additive (0.0–2.0 wt%) and sintered at various temperatures. As can be seen there is an optimum additive amount for each sintering temperature, Fig. 6.

As mentioned in former section variation of the initial permeability around the optimum sintering temperature, is subjected to two parameters, enhancement in grain growth and reduction in intragranular porosities. The results show that addition of additive not only enhances initial permeability, but reduces the optimum sintering temperature. As can be seen at each temperature initial permeability of the x = 0.65 sample with 1 wt% additive is mostly maximum and the highest value is 492 for Ts = 1280 °C.

**Figure 7:**
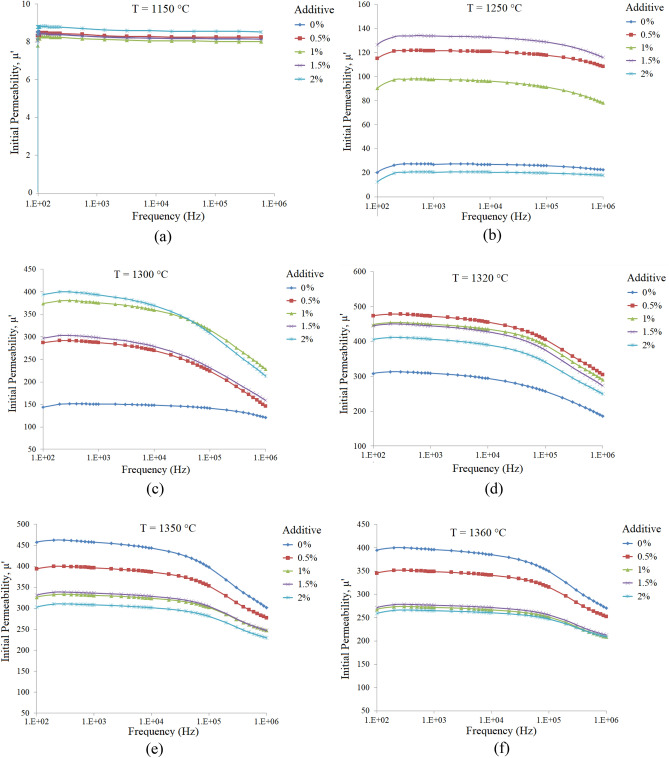
Initial permeability spectra of the Ni_0.3_Zn_0.7_Fe_2_O_4_ samples with additive (0.0–2.0 wt%) and sintered at various temperatures: (**a**) 1150, (**b**) 1250, (**c**) 1300, (**d**) 1320, (**e**) 1350 and (**f**) 1360 °C.

**Figure 8:**
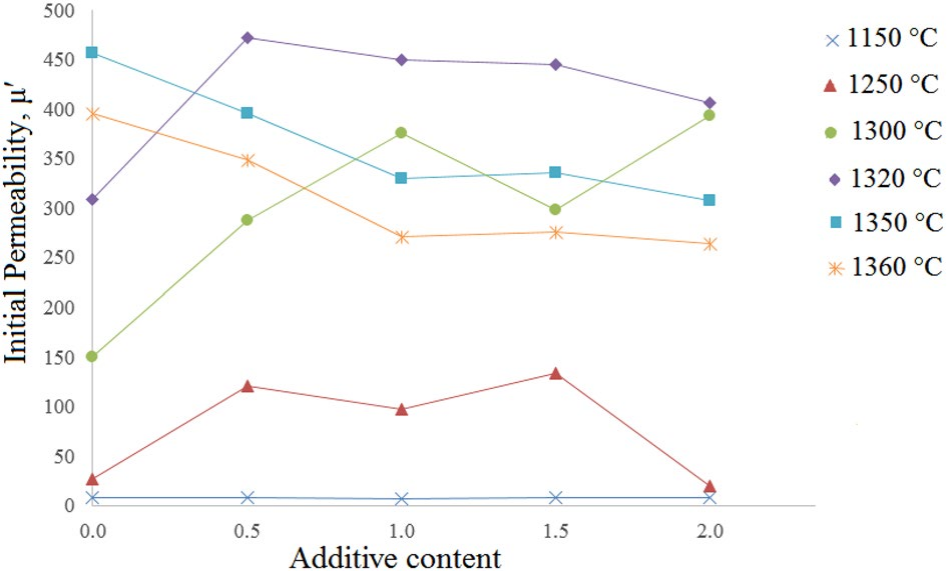
The variation of initial permeability (@ 1 kHz) of the Ni_0.35_Zn_0.65_Fe_2_O_4_ samples with respect to additive contents, sintered at various temperatures as labeled on each curve (The lines were added to guide eye).

Figure 7a–f show the initial permeability spectra of the x = 0.70 samples with additive (0.0–2.0 wt%) and sintered at various temperatures. As can be seen there is an optimum additive amount for each sintering temperature, Fig. 8.

Figure 7a (T_s_ = 1150 °C) shows that although permeabilities of the samples are low (< 10), but they are very close to each other and relatively independent of frequency. By increasing the sintering temperature to 1250 °C, additive clearly shows its marvels effect, so the permeability increases drastically more than one order, it increases from about 8 to 134 for 1.5 wt% additive content. As can be seen by increasing sintering temperature, maximum permeability of 478 is achieved for Ts = 1320 °C and 0.5 wt% additive. A comparison between these results with those obtained former (Ts = 1280 °C, 492 and 1.0 wt% additive for x = 0.65) shows that there is a tight coupling between composition, sintering temperature and additive content. Therefore, optimum additive contents are 1.0 and 0.5 wt% for x = 0.65 and 0.70, respectively and this is related to the microstructure and the effect of additive on it.

The effects of additives on the microstructure of Ni–Zn ferrites can be explained by additives segregations on the grain boundaries. By the way, immigration of the grain boundaries is impeded and then preventing further crystal growth, which leads to lesser intragranular pores and absence of very large grains^43^. Permeability spectra of the x = 0.65 samples with different additive contents which are sintered at various temperatures demonstrated that permeability is approximately constant at lower frequencies (< 100 kHz). The same behavior is seen for the x = 0.70 samples, except that the ferrimagnetic resonance occurs at lower frequencies, around 10 kHz.

**Figure 9:**
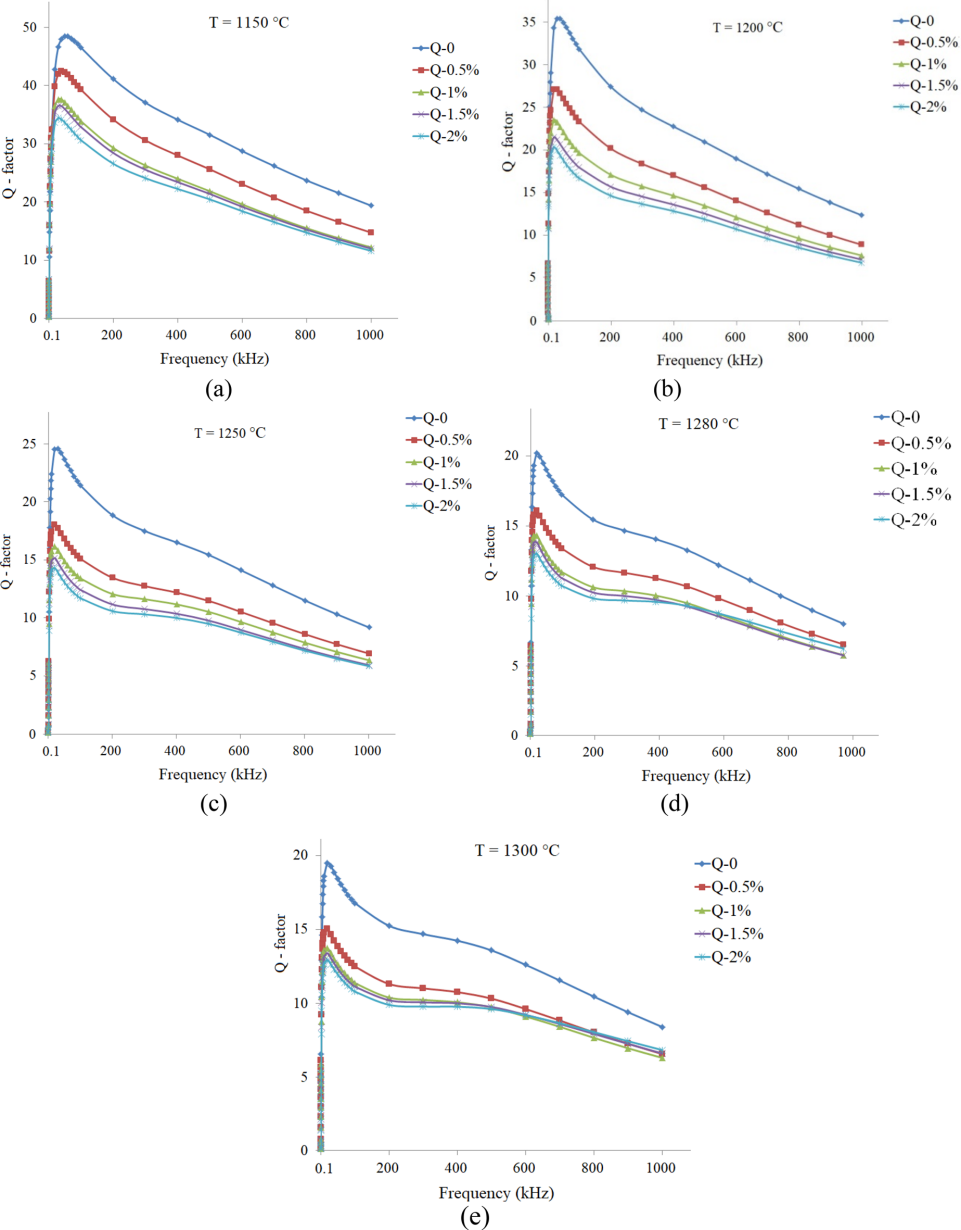
Q-factor spectra of the Ni_0.35_Zn_0.65_Fe_2_O_4_ samples with additive (0.0 up to 2.0 wt%) and sintered at various temperatures: (**a**) 1150, (**b**) 1200, (**c**) 1250, (**d**) 1280 and (**e**) 1300 °C.

**Figure 10:**
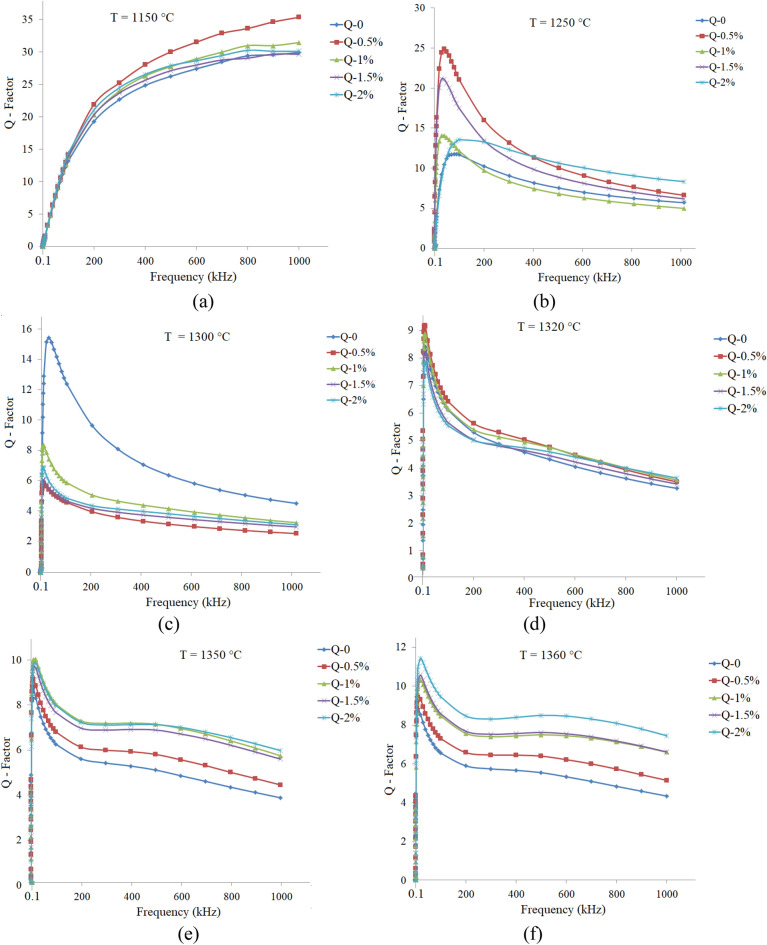
Q-factor spectra of the Ni_0.3_Zn_0.7_Fe_2_O_4_ samples with additive (0.0 up to 2.0 wt%) and sintered at various temperatures: (**a**) 1150, (**b**) 1250, (**c**) 1300, (**d**) 1320, (**e**) 1350 and (**f**) 1360 °C.

Q-factor spectra of the samples with different amounts of additive that sintered at various temperatures are shown in Figs. 9a–e and 10a–f for the x = 0.65 and 0.70, respectively. As can be seen, Q-factors have sharp peaks, except that related to x = 0.70 sintered at 1150 °C. The highest Q-factor values are belonged to the additive free samples, which may be related to smaller grain sizes and the lesser defects within the grains than those samples sintered at higher temperatures.

## Conclusion

In this work nickel zinc ferrites (Ni_1−x_Zn_x_Fe_2_O_4_, x = 0.65 and 0.70) were prepared via conventional solid-state reaction method. Also an equiponderant mixture of Bi_2_O_3_, CaO, CeO_2_, SiO_2_, Al_2_O_3_, Y_2_O_3_ and nanotitania was calcined at 1100 °C and named multi compound calcined additive (MCCA). The MCCA causes to decrease the sintering temperature and increase the permeability remarkably in comparison with noncalcined additives. This is due to partly segregation of the calcined additive on the grain boundaries, which influences the grain boundaries’ resistivity and in addition diffusion of the other parts in the spinel lattice leads to improvement magnetic properties. It can be concluded the MCCA added Ni_0.35_Zn_0.65_Fe_2_O_4_, is suitable for application in the switching power supplies.

## Data Availability

All data generated or analyzed during this study are included in this published paper.
